# Alumina-on-alumina total hip replacement for femoral neck fracture in healthy patients

**DOI:** 10.1186/1471-2474-12-32

**Published:** 2011-02-01

**Authors:** Giuseppe Solarino, Andrea Piazzolla, Claudio M Mori, Lorenzo Moretti, Silvio Patella, Angela Notarnicola

**Affiliations:** 1 Orthopaedic Department, Università degli Studi di Bari, Italy

## Abstract

**Background:**

Total hip replacement is considered the best option for treatment of displaced intracapsular fractures of the femoral neck (FFN). The size of the femoral head is an important factor that influences the outcome of a total hip arthroplasty (THA): implants with a 28 mm femoral head are more prone to dislocate than implants with a 32 mm head. Obviously, a large head coupled to a polyethylene inlay can lead to more wear, osteolysis and failure of the implant. Ceramic induces less friction and minimal wear even with larger heads.

**Methods:**

A total of 35 THAs were performed for displaced intracapsular FFN, using a 32 mm alumina-alumina coupling.

**Results:**

At a mean follow-up of 80 months, 33 have been clinically and radiologically reviewed. None of the implants needed revision for any reason, none of the cups were considered to have failed, no dislocations nor breakage of the ceramic components were recorded. One anatomic cementless stem was radiologically loose.

**Conclusions:**

On the basis of our experience, we suggest that ceramic-on-ceramic coupling offers minimal friction and wear even with large heads.

## Background

Fractures of the femoral neck (FFN) are very common in orthopaedic practice. When an intracapsular lesion occurs, it may be treated by either reduction and internal fixation, which preserves the femoral head, or by replacement of the femoral head with an arthroplasty. The aim of both operations is to restore the patient's pre-injury function as quickly as possible.

Garden's classification of proximal femoral fractures is the most widely used, and is useful as it is both simple and predicts the development of AVN [1].

Garden stage I : undisplaced incomplete, including valgus impacted fractures,

Garden stage II : undisplaced complete fracture,

Garden stage III : complete fracture, incompletely displaced,

Garden stage IV : complete fracture, completely displaced.

In view of the much higher failure rate after internal fixation - leading to increased suffering for these patients - primary arthroplasty stands out as the best method for displaced Garden III and IV FFN [2].

When a total hip arthroplasty (THA) is performed, the surgeon must take into account dislocation of the implant as a possible complication. This is claimed to be more frequent after a hip fracture treated with the posterior surgical approach, in elderly patients with soft-tissue laxity [3].

A report by the Norwegian Arthroplasty Registry underlines the fact that the femoral head size is a risk factor for total hip luxation, and that 28 mm heads require revision significantly more often than 32 mm, and 26 mm heads more often than 30 mm heads. The preoperative diagnosis, i.e. femoral neck fracture, was also an important factor affecting the revision rate due to luxation [4].

We designed a retrospective cohort clinical study to evaluate the results of THAs performed for displaced FFN, using a 32 mm alumina-alumina (Al-Al) coupling.

## Methods

The study was approved by the Local Ethical Committee and was carried out in compliance with the Helsinki Declaration.

From March 1996 to March 2006, 782 hip arthoplasties were performed at our Institution.

Of these 421 were endoprostheses and 361 arthroprostheses, 244 being elective surgery for coxarthrosis, osteonecrosis etc., and 117 performed for fracture of the femoral neck. Of these 117, 32 mm Alumina-Alumina coupling was applied in only 35, while in the remaining 82 different sizes and coupling were adopted. In this study we selected only the 32 mm alumina-on-alumina total hip replacements which were performed for femoral neck fractures in patients without co-morbidities nor mental disease and aged <75 years, ensuring a long follow-up.

During this 10-year period, 35 displaced intracapsular fractures of the upper femur (31 females and 4 males) were treated with an alumina-alumina hip replacement. Diagnosis was made on an anteroposterior view of the pelvis and a lateral radiograph of the involved hip (tables 1 and 2). Of these fractures, 17 were classified as Garden III and the remainder as Garden IV (table 2).

**Table 1:** Patients age, sex (F/M), side of the surgery, type of stem, (L = long; M = medium; S = short), cup size inclination and Garden class at the time of implantation

Id. code	Age (years)	Sex	side	Type	Neck	Cup size (mm)	Cup Inclination (degree)	Screws	Garden (At the time of surgery)
**1**	66	F	RIGHT	OSTEAL	M	54	45	YES (N.2)	3

**2**	64	F	RIGHT	ANATOMIC	L	54	44	NO	3

**3**	66	F	LEFT	ANATOMIC	M	52	44	NO	4

**4**	59	F	RIGHT	ANATOMIC	M	48	44	NO	4

**5**	69	M	RIGHT	ANATOMIC	L	54	45	YES (N.2)	4

**6**	66	F	RIGHT	ANATOMIC	L	54	46	NO	4

**7**	55	F	RIGHT	OSTEAL	S	52	46	YES (N.2)	3

**8**	61	F	RIGHT	ANATOMIC	M	50	48	NO	4

**9**	67	F	LEFT	ANATOMIC	L	50	44	NO	4

**10**	69	F	RIGHT	ANATOMIC	S	50	43	YES (N.2)	4

**11**	74	M	LEFT	ANATOMIC	L	56	43	NO	4

**12**	69	F	RIGHT	ANATOMIC	M	50	44	YES (N.2)	4

**13**	71	F	RIGHT	OSTEAL	L	48	43	NO	3

**14**	73	F	RIGHT	ANATOMIC	L	50	46	YES (N.2)	4

**15**	66	F	LEFT	ANATOMIC	S	54	43	YES (N.2)	4

**16**	58	F	LEFT	OSTEAL	L	50	44	NO	3

**17**	73	F	LEFT	ANATOMIC	M	54	43	NO	4

**18**	60	F	LEFT	OSTEAL	S	60	46	YES (N.2)	3

**19**	72	F	RIGHT	OSTEAL	L	58	43	NO	3

**20**	64	F	LEFT	OSTEAL	L	52	46	NO	3

**21**	61	M	RIGHT	OSTEAL	M	54	45	NO	3

**22**	72	F	LEFT	OSTEAL	M	50	43	YES (N.2)	3

**23**	61	M	LEFT	OSTEAL	M	52	45	NO	3

**24**	72	F	LEFT	OSTEAL	M	52	43	NO	3

**25**	73	F	LEFT	OSTEAL	M	50	46	YES (N.2)	3

**26**	63	F	RIGHT	OSTEAL	M	50	44	YES (N.2)	3

**27**	53	F	LEFT	MULTICON	M	50	43	NO	4

**28**	69	F	LEFT	MULTICON	S	50	44	YES (N.2)	4

**29**	72	F	RIGHT	MULTICON	L	52	43	YES (N.2)	3

**30**	68	F	LEFT	MULTICON	M	54	44	NO	4

**31**	47	F	RIGHT	OSTEAL	M	54	45	NO	3

**32**	62	F	LEFT	MULTICON	S	50	43	NO	4

**33**	75	F	RIGHT	OSTEAL	M	52	43	NO	3

**34**	71	F	RIGHT	MULTICON	S	50	47	YES (N.2)	4

**35**	69	F	RIGHT	MULTICON	M	54	44	YES (N.2)	4

**MEAN**	66	4 M/31 F	16 LEFT/19 RIGHT			52.1	44.3		

**SD**	6.6					2.6	1.3		

**Table 2:** Garden classification and Harris Hip Score (HHP) at the different Follow-up (FU) times

Id. code	HHS (At 1 month after surgery)	HHS (At last Follow-Up)	Months of last FU
**1**	100	100	144

**2**	96	96	137

**3**	100	100	126

**4**	94	94	125

**5**	96	96	122

**6**	100	100	115

**7**	100	100	112

**8**	91	91	110

**9**	100	100	108

**10**	100	100	106

**11**	100	100	104

**12**	100	100	104

**13**	100	100	101

**14**	100	100	100

**15**	100	100	93

**16**	100	100	89

**17**	100	100	88

**18**	90	90	86

**19**	96	96	78

**20**	90	90	77

**21**	100	100	73

**22**	86	86	73

**23**	100	100	66

**24**	94	94	65

**25**	100	100	61

**26**	100	100	54

**27**	100	100	46

**28**	98	98	42

**29**	96	96	35

**30**	98	100	34

**31**	87	87	28

**32**	97	100	26

**33**	98	100	26

**34**	98	100	25

**35**	100	100	24

**MEAN**	97.3	97.5	80

**SD**	4	4.1	36

Median patients age at the time of surgery was 66 years (range 47-75 years). All the operations (involving the right side in 19 cases, the left in 16) were primary procedures (none previously treated with internal fixation), performed in a conventional turbulent flow theatre, via the direct lateral approach described by Hardinge [5] to expose the hip joint.

The press-fit cup, hammered into a 2 mm under-reamed acetabulum, consisted of a pure titanium core with a titanium alloy mesh: it is grossly hemispherical in shape (with polar flattening and circumferential gutters, and a Triradius-M Cup), with one hole on the apex for the liner, inserted by conical sleeving. This cup was always combined with a 32 mm femoral head. Both the inlay and the head were made of dense polycrystalline surgical-grade alumina (Al2O3).

**Figure 1:**
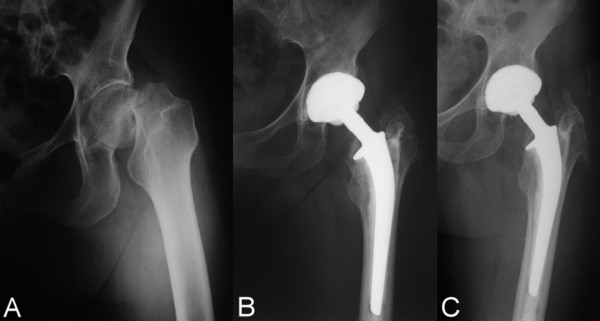
**hybrid THA preoperatively (A), postoperatively (B) and at 9 (C) years of follow-up**.

**Figure 2:**
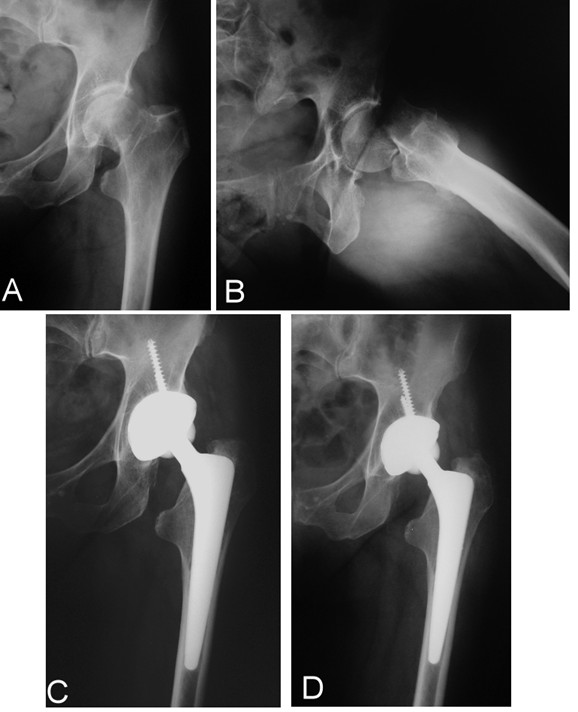
**cementless THA preoperatively (A-B), at 1 (C) and 4 (D) years postoperatively**.

Two additional screw fixations were fitted in 15 cases, in the two further holes of the shell. The mean cup inclination was 44°34' post-operatively (range 43°-48°). The alumina head was anchored via a Morse taper on three different femoral components made of anodized Titanium-alloy (TiAl6V4): in 15 cases (42.85%) a smooth, collared stem ("Osteal") was cemented in (Figure 1), and in 20 cases (57.15%) two collarless cementless stems: in 13 an anatomical, smooth stem with a trochanteric wing and a medial porous coating mesh was applied in the proximal part ("Anatomic"), in 7 a straight, three-dimensionally tapered wedge with anti-rotational ribs in the proximal part and a rough blasted surface ("Multicone") was applied (Figure 2). All of them had a 12-14 morse cone. All the components were manufactured by Ceraver (Ceraver Osteal, Roissy, France).

In the hybrid implants, a distal cement restrictor was used, the medullary canal was cleaned with saline lavage and an injection gun was employed, together with digital pressurization of the cement.

Perioperative care was the same for all patients: thromboembolic (heparin administration and compression stockings) and antibiotic prophylaxis, passive motion exercises with the assistance of a therapist immediately after the operation, then leaving patients free to walk with two supports after 3 days, for about 6 weeks. Thereafter, full weight-bearing was usually authorized.

Clinical and radiographic follow-up was performed at six weeks, three months, six months, and one year after the operation and yearly thereafter. Serial anteroposterior radiographs of the pelvis were analyzed by the same observer (AP), who was not involved in the operations. On the AP pelvis X-ray we found the no presence of tilting by 2° or more and/or a penetration of 2 mm or more.

Harris hip ratings [6] were determined at each follow-up examination: a Harris hip score of 90 points or more was defined as an excellent outcome; 80 to 89 points, a good outcome; 70 to 79 points, a fair outcome; and less than 70 points, a poor outcome.

Loosening of the socket was defined as a cup migration by more than 3 mm, angular rotation exceeding 3°, or a continuous radiolucent line wider than 2 mm, according to the zones described by DeLee and Charnley [7].

As to the femoral aspects, the parameters investigated included subsidence of the stem, calcar resorption and progression of radiolucent lines according to the 7 zones described by Gruen [8]: loosening of the cemented stem was defined as a migration by more than 3 mm or a continuous radiolucent line wider than 2 mm. Assessment of the mechanical implant stability completed the investigation of signs of osseointegration, as supported by Engh [9].

## Results

### Clinical results

At a mean follow-up of 80 months (range 24-144 months), 33 hips have been clinically and radiologically reviewed. Meanwhile, two patients died of causes unrelated to the operation (malignant tumors); these two cases were analyzed on their previous clinical history and radiographic images.

At the last follow-up, the Harris hip score had increased to a mean value of 97.5 (SD 4.1) median 100) (range 86-100): 33 were considered excellent, 2 good, while none were judged fair or poor.

### Radiological results

All the cups were well fixed at the latest follow-up, without migration and/or tilting.

None of the implants needed to undergo revision for any reason. Two cemented stems had a non progressive radiolucent line of <1 mm at the cement-bone interface in zones 1 and 7, none of them had migrated or tilted; one cementless anatomical stem showed evidence of mobilization defined as aseptic loosening (i.e. sinking by 2 mm and pedestal formation) raising no clinical concern at 9-year follow-up. All the cementless straight stems were judged stable, but some of them showed thinning <2 mm of the calcar femorale; pedestal formation was never observed.

On the AP pelvis X-ray no presence of tilting by 2° or more or penetration by 2 mm or more was observed.

### Complications

Ceramic wear was undetectable. None of the implants underwent any dislocation and none of the ceramic components broke down. No patient showed stem anteversion. No infectious complications developed. A transient squeaking sensation was reported by one patient.

## Discussion

The strong point of this work is that it demonstrates that the use of alumina-on-alumina implants for total hip replacement yields satisfactory results in the medium term in young patients with no co-morbidities and in good mental health. The weak points are the absence of a control group, the small sample size and the non homogeneous follow-up period.

In the treatment of a displaced intracapsular FFN, the surgeon needs to weigh up reduction and internal fixation or hip replacement as the surgical options; the former features a shorter surgery time, less operative blood loss, no need for blood transfusion or risk of deep wound infection, but arthroplasty has a lower re-operation rate [10]. Since the fracture is often a direct result of osteoporosis, this risk of higher failure must be taken into account especially in active elderly patients [11], or in patients with chronic diseases [12]. Re-operations are reported to be necessary in 2 - 8% of patients after THA, and in 14 - 53% after internal fixation [2]. At a 4-year follow-up evaluation, complication and reoperation rates were ten times lower using THA [13]. Therefore, nowadays there can be no doubt that total joint arthroplasty is the most clinically effective and most durable procedure in these situations [14]; it is shown to be the best method even when evaluating the accumulated costs of each method during the first 2 years after the fracture [15].

Despite the efficacy of THA, complications can occur which yield poor functional outcomes in a subset of patients. The 90-day complication rate after primary THA was 3.8%: the dislocation rate (1.4%), and mortality rate 0.68%. Apart from these, the rates of infection, thromboembolic disease (including pulmonary embolism and deep venous thrombosis), neurovascular injury, perioperative fracture, and revision surgery were all below 1%. Increased age was associated with a higher risk of a short-term complication. The presence of complicated diabetes increases the risks of mortality and infection [16]. Late complications include prosthetic loosening, displacement, metallosis, osteonecrosis and heterotopic bone formation in 2-5% of patients [17,18].

When a metal backing is used in acetabular cup design, it generates high stress peaks around the acetabular rim, causing peri-acetabular bone loss [19].

There is no clear consensus as to the optimal management of patients aged between sixty and eighty years [20], and even less for young active patients, but it must be borne in mind that if internal fixation is unsuccessful and revision of a THA is required, the risk of early complications is higher and hip function may be poorer than if arthroplasty had been performed as a primary procedure [21].

When a THA is performed, the risk of dislocation should be taken into account: it is higher both after a hip fracture and in elderly patients, because of the poor muscular strength and the attempts made to regain the pre-injury full range of motion [3,4].

In a retrospective work on 42,987 primary operations, Bystrom S et al. [4], demonstrated that the femoral head size was an important risk factor for prosthesis luxation: 22 mm heads performed equally well or better than 28 mm heads, but 28 mm heads required revision four times more often than 32 mm ones.

Heads larger than 28 mm can be used if we move to hard-on-hard couplings: ceramic-on-ceramic are attractive alternative bearing surfaces that have been reported to eliminate or reduce the problems related to polyethylene wear debris. Because of its sliding characteristics (lower frictional torque, better wettability, less reactive wear particles than polyethylene), it is possible to increase the femoral head diameter, according to the Low Frictional Torque Arthroplasty theory.

A 32 mm head grants a wider range of movement than a 28 mm head [22]. This is true of any type of coupling in the very short term but the very low wear with ceramic-on-ceramic avoids penetration of the head into the liner, as occurs with polyethylene, ensuring that this optimal range of movement is long-lasting. When a liner develops wear, the centre of rotation migrates centrally and/or cranially, and the deeper the head, the more restricted the range of movements becomes (7° are lost for each millimetre of penetration); in fact, sometimes late dislocation can be the first clinical sign of wear [3]. Nevertheless, in our clinical practice the incidence of failure is low and other solutions involving the use of polyethylene have proven equally valid.

Although literature reports of ceramic-on-ceramic 32 mm coupling describe a high risk of fracture and squeaking, as well as a more technically demanding procedure, our data confirm that this type of coupling can protect the hip from dislocation, both postoperatively and at mid-term follow-up.

Squeaking, defined as a reproducible squeaking, clicking, or grating sound, is an underestimated problem that is recurrent in ceramic-on-ceramic THA. It has been hypothesized that the sound is caused by a short neck length of the femoral implant [23].

We did not observe any fracture of the components: this can be explained by the precise manufacture and contact surface geometry, including optimal clearance (in our series cup, liner, head and stems were all produced by the same manufacturer) and because of the fact that resistance to fracture is increased if a 32 mm head is used,. Santavirta S [24] has stated that for the currently available ceramic products, the component fracture risk is almost nonexistent even with a 28 mm head, as shown in clinical investigations at 4 and 5 years [25,26].

Further advantages of both strength and articularity are obtained with an even larger diameter: the rates of dislocation, in the first three months after surgery, are 0.88% for 36 mm and 4.64% for 28 mm, respectively; these percentages fall to 0% (0 cases out of 16) versus 10% (3 cases out of 30) in patients operated for a femoral neck fracture [27].

Finally, we did not observe any failure of the cup, defined as tilting by 2° or more and/or a penetration of 2 mm or more, shown on the AP pelvis X-ray. It is thought that the titanium shell may act as a shock absorber between the very rigid alumina and the probably porotic bone, solving the problem of socket fixation reported when a bulky alumina cup was cemented into the acetabulum [28].

We are aware that the greater cost of ceramic-on-ceramic than ceramic-polyethylene coupling is a problem. Nevertheless, if clinical studies of larger cohorts demonstrate a better survival, they will justify this surgical choice.

## Conclusions

In our experience, total hip arthroplasty stands out as a good method of treatment of intracapsular displaced fractures of the femoral neck. Ceramic-on-ceramic coupling offers minimal friction and wear even with heads larger than 28 and 22.2 mm. The use of a ball measuring 32 mm or more complies with sir John Charnley's theory and allows a wider range of movement of the artificial joint, that persists over the years, protecting the hip from early or late dislocation.

## Competing interests

The authors declare that they have no competing interests.

## Authors' contributions

GS and AP gave substantial contributes in the drafting the manuscript and in the revising it for the intellectual contents. LM and SP participated in the acquisition of data of case reports. BM gave substantial contributions to interpretation of literature review. CM and AN participated in the analysis and interpretation of data, and reviewed the manuscript. All authors read and approved the final manuscript.

## Pre-publication history

The pre-publication history for this paper can be accessed here:

http://www.biomedcentral.com/1471-2474/12/32/prepub
